# The girdles of the oldest fossil turtle, *Proterochersis robusta*, and the age of the turtle crown

**DOI:** 10.1186/1471-2148-13-266

**Published:** 2013-12-06

**Authors:** Walter G Joyce, Rainer R Schoch, Tyler R Lyson

**Affiliations:** 1 Department of Geosciences, University of Tübingen, Hölderlinstr. 12, 72074 Tübingen, Germany; 2 Department of Geosciences, University of Fribourg, 1700 Fribourg, Switzerland; 3 Staatliches Museum für Naturkunde Stuttgart, 70191 Stuttgart, Germany; 4 Department of Vertebrate Zoology, National Museum of Natural History, Smithsonian Institution, Washington DC, WA 20560, USA

## Abstract

**Background:**

*Proterochersis robusta* from the Late Triassic (Middle Norian) of Germany is the oldest known fossil turtle (i.e. amniote with a fully formed turtle shell), but little is known about its anatomy. A newly prepared, historic specimen provides novel insights into the morphology of the girdles and vertebral column of this taxon and the opportunity to reassess its phylogenetic position.

**Results:**

The anatomy of the pectoral girdle of *P. robusta* is similar to that of other primitive turtles, including the Late Triassic (Carnian) *Proganochelys quenstedti*, in having a vertically oriented scapula, a large coracoid foramen, a short acromion process, and bony ridges that connect the acromion process with the dorsal process, glenoid, and coracoid, and by being able to rotate along a vertical axis. The pelvic elements are expanded distally and suturally attached to the shell, but in contrast to modern pleurodiran turtles the pelvis is associated with the sacral ribs.

**Conclusions:**

The primary homology of the character “sutured pelvis” is unproblematic between *P. robusta* and extant pleurodires. However, integration of all new observations into the most complete phylogenetic analysis that support the pleurodiran nature of *P. robusta* reveals that this taxon is more parsimoniously placed along the phylogenetic stem of crown Testudines. All current phylogenetic hypotheses therefore support the basal placement of this taxon, imply that the sutured pelvis of this taxon developed independently from that of pleurodires, and conclude that the age of the turtle crown is Middle Jurassic.

## Background

Turtles are one of the most enigmatic groups of living vertebrates and many questions remain unanswered regarding the origin of the group and the age of the crown clade. Whereas much has recently been written on the origin of turtles [1-7] and significant progress has been made on the origin of their unique body plan [8-11], the debate is still ongoing regarding the age of the crown clade and the origin of the two main extant turtle lineages: pleurodires and cryptodires. For instance, a series of recent papers have explored whether the Early Jurassic turtle *Kayentachelys aprix* is best interpreted as the oldest known stem cryptodire [12,13] or a stem turtle [14-16]. However, these different interpretations imply a significantly different age of the turtle crown, which in return informs and/or conflicts with current molecular clock studies [5,17-19].

The concurrent debate regarding the phylogenetic placement of the oldest known shelled amniote, *Proterochersis robusta* from the Late Triassic (Middle Norian) of Germany, is of equal importance. This taxon has traditionally been thought to have a pelvis that is sutured to the inside the shell (i.e., a “sutured pelvis”) and to therefore be an early stem pleurodire, as this is traditionally believed to be an unambiguous apomorphy of the group [12,20,21]. However, others have argued that the sutured pelvis originated twice [22] or have even doubted the presence of this character in this taxon [14]. We here present a newly prepared specimen of *P. robusta* from the Late Triassic of Baden-Württemberg, Germany that not only exhibits all details of the pelvis, but also of the pectoral girdle and part of the vertebral column. The specimen is of particular importance because it helps clarify the orientation of the scapula among basal turtles, confirms the unambiguous presence of a sutured pelvis in *P. robusta*, and provides an abundance of other character information that further corroborates its basal position of this taxon along the turtle stem lineage.

## Methods

SMNS (Staatliches Museum für Naturkunde Stuttgart) 17757 was collected by a forest worker in 1933 between the villages of Klaffenbach and Althütte, about 40 km WNW of Stuttgart, Baden-Württemberg, Germany. The specimen was shortly thereafter acquired by the Royal Natural History Collection of Württemberg (the precursor of SMNS), but it appears to have been completely ignored by scientists and remained undescribed to date. Although the precise locality is not preserved, the fossil certainly originated from the Lower Stubensandstein, which falls within the basal part of the k5 sandstone unit of the Keuper (Löwenstein Formation) and corresponds to the Middle Norian (Alaunian), ca. ~212-210 Ma [23]. All known specimens of *P. robusta*, including the holotype, were collected in the broader vicinity of SMNS 17757 (i.e., the region between the Murrhardt and Rems rivers) and from the same stratigraphic layers (pers. comm. Dieter Seegis), and the attribution of SMNS 17757 to *P. robusta* is unambiguously supported by the presence of a high-domed carapace, two pairs of abdominal scutes, and the morphology of the pelvis [20]. SMNS 17757 suffered extensive damage during recovery and most of the carapacial and plastral bones are missing, which is likely the primary reason why this specimen was ignored for so long. However, given that parts of the girdles and vertebral column were protruding from the remaining steinkern, preparation was initiated in recent years resulting in the exposure of the girdles and portions of the vertebral column associated with the shell. A series of photographs were taken by the preparator during preparation using a low-budget point and shoot camera that document the position at which various bones were found prior to their removal from the block (see Additional file 1: Figure S1).

The phylogenetic position of *P. robusta* has been resolved to be at the very base of the turtle lineage by multiple analyses in recent years [14,22,24-26], but opposition is fierce and some still favour placing this taxon at the base of the pleurodiran lineage [12,27,28]. To test the impact of the novel morphological insights provided by this study, we modified the analysis of Gaffney et al. [12], which is the most recent global analysis to advocate the pleurodiran affinities of this taxon. The following modifications were undertaken:

1) The Late Triassic proto-turtle *Odontochelys semitestacea*[29] was added to the matrix based on personal observations of the paratype (Institut for Vertebrate Paleontology and Paleoanthropology V13240) by WGJ and TRL.

2) The scoring of the Early Jurassic stem-turtle *Kayentachelys aprix* was modified following Joyce and Sterli [16]. All “problem characters” were scored derived (see [16]), thereby favouring the cryptodiran affinities proposed for this taxon [12].

3) The composite taxon “Megapleurodira” was split into the Late Jurassic stem-pleurodire *Platychelys oberndorferi* and crown group Pleurodira based on personal observations of the relevant material by WGJ and TRL. The scoring of crown Pleurodira differs from that of Megapleurodira in the scoring of seven characters: 65 (1, not 0&1, i.e., cervical vertebrae formed); 70 (1&2, not 0&1&2, i.e., cervical vertebrae pro- or opisthocoelous); 76 (1&2, not 0&1&2, i.e., 8th cervical procoelous or biconvex); 87 (1, not 0&1, i.e., first thoracic reduced); 94 (2, not 1, i.e., supramarginals absent); 97 (1, not 0&1, i.e., plastral buttresses reach costals); 109 (0, not ?, i.e., pectoral scute does not overlap onto entoplastron).

4) The scoring of *Proterochersis robusta* was updated based on the new observations presented herein. In addition to replacing numerous missing scorings, the following corrections were undertaken for *P. robusta*: character 86 (?, not 1, we cannot replicate the meaning of this character and therefore score it as unknown); character 91 (?, not 1, i.e., it is unknown if the 10^th^ thoracic rib contributes to the sacrum); and 99 (1 or 2, not 0, i.e., the dorsal epiplastral processes does not contact the nuchal dorsally).

5) We added a character state to character 103 (i.e., 0 = two pair of mesoplastra present, 1 = one pair of mesoplastra present, 2 = mesoplastra absent). See Additional file 2 for complete character/taxon matrix.

A maximum parsimony analyses was performed using PAUP 4.0b10 [30]. All characters were left unordered and unweighted, minimum branch length were set to collapse if branch lengths equalled zero, and the most parsimonious solution was sought using 1000 randomly seeded heuristic searches, thereby closely replicating the analysis of Gaffney et al. [12].

## Results

### Pectoral girdle

**Figure 1:**
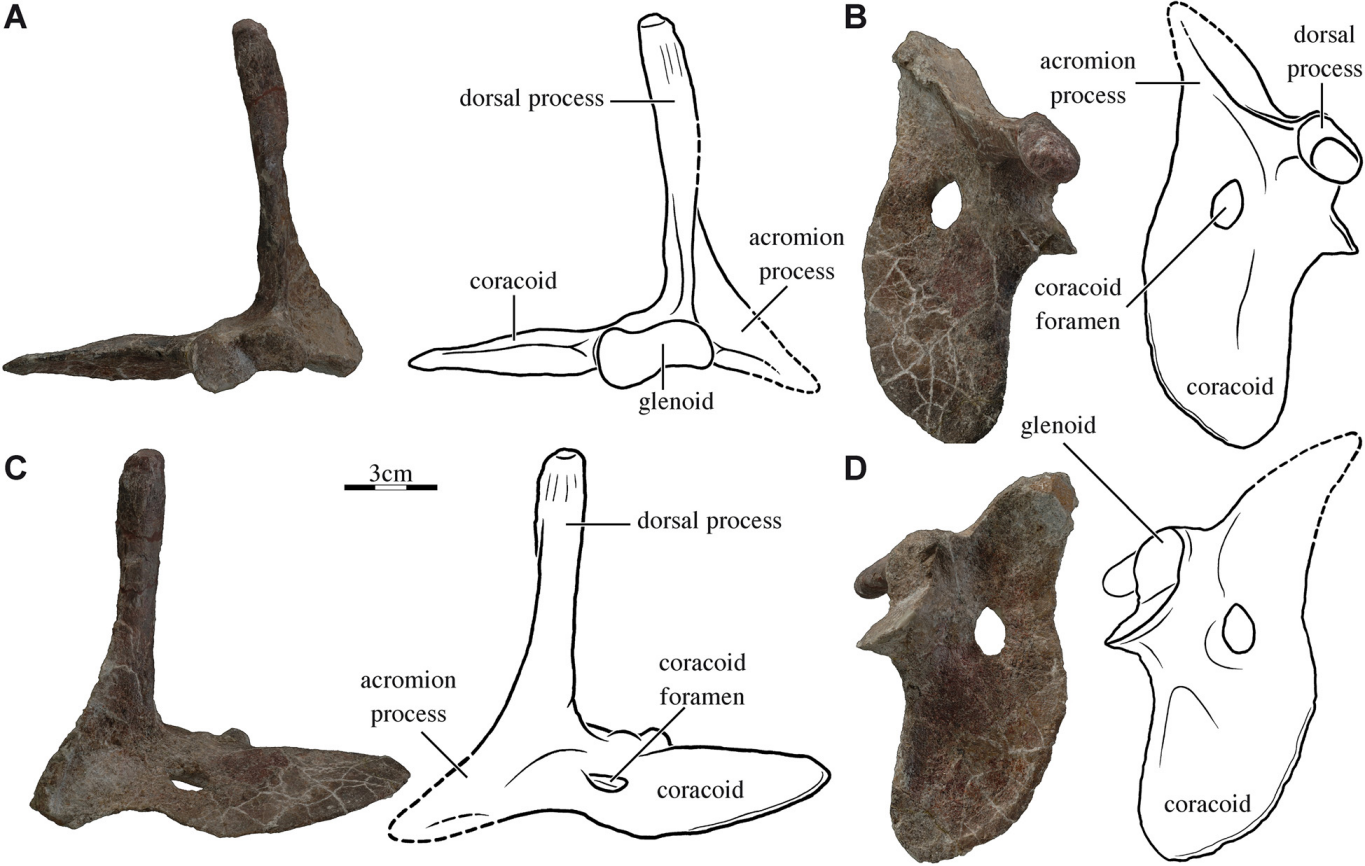
**SMNS 17757, *Proterochersis robusta*, right scapulacoracoid, Late Triassic (Norian) Löwenstein Formation of Baden-Württemberg, Germany. (A)** Photograph and illustration in right lateral view. **(B)** Photograph and illustration in dorsal view. **(C)** Photograph and illustration in medial view. **(D)** Photograph and illustration in ventral view.

The dorsal process of the scapulacoracoid is elongate and striated distally (Figure 1). The acromial process is only half the length of the dorsal process, is slightly curved distally, and connected to the dorsal process, the glenoid, and the coracoid by bony ridges. The glenoid is fused, peanut-shaped, lacks a distinct neck, and consists of a flat facet formed by the coracoid and a flat facet formed by the scapula that are arranged at an angle of 120 degrees relative to one another (Figure 1B). The coracoid is a broad, flattened blade and a distinct coracoid foramen is present. The right and left scapulacoracoids were removed from the block during preparation, but the scapular processes were oriented vertically (see Additional file 1: Figure S1A, C), the distal end of the acromion was only separated by a small gap from the plastron, and the coracoid blades were oriented horizontally essentially mirroring the condition seen in all extant turtles (see Additional file 1: Figure S1B).

### Pelvic girdle

**Figure 2:**
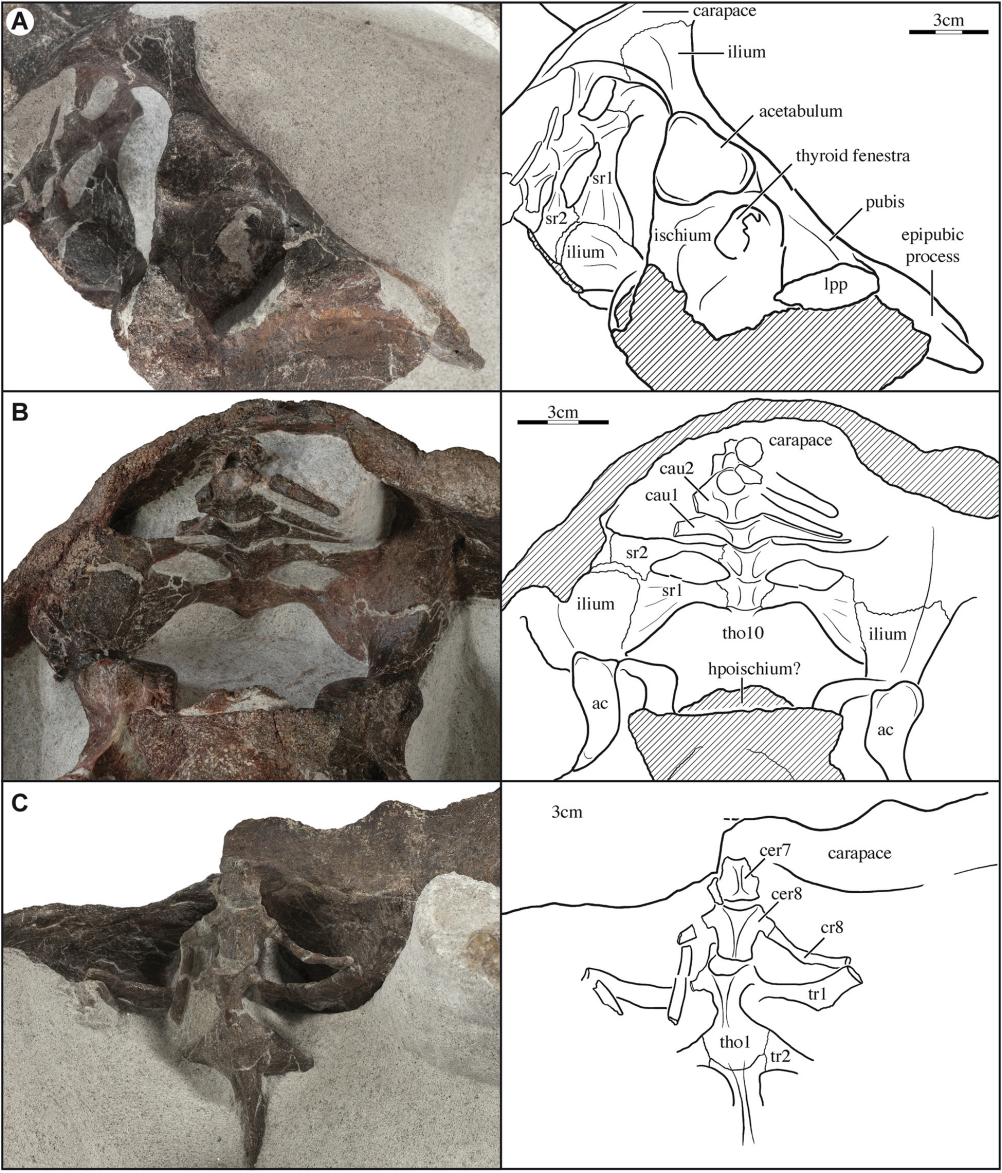
**SMNS 17757, *Proterochersis robusta*, Late Triassic (Norian) Löwenstein Formation of Baden-Württemberg, Germany. (A)** Photograph and illustration of pelvic girdle in oblique right ventrolateral view. **(B)** Photograph and illustration of sacrum in oblique posteroventral view. **(C)** Photograph and illustration of posterior cervical column and anterior thoracic column in ventral view. *Abbreviations*: ac = acetabulum; cau = caudal vertebra; cer = cervical vertebra; cr = cervical rib; lpp = lateral pubic process; sr = sacral rib; tho = thoracic vertebra; tr = thoracic rib. Shaded areas represent damaged bone surfaces.

The elements of the pelvic girdle are fully fused with one another and it is therefore not possible to assess their relative contributions to the acetabulum (Figure 2A, B). The acetabulum is oriented laterally and has the outline of a rounded triangle.

The ilium has a short neck that expands distally to form a broad and rounded sutural contact with the carapace. However, in contrast to extant pleurodires, where the carapace received the ilium via a facet, the carapace is thickened at the articulation site to form a broad descending process (Figure 2A, B).

The right pubis is disarticulated from the plastron and it is therefore possible to study the articular process in detail. The pubis articulates with the plastron along a distally expanded, anteroposteriorly elongated process. The distal end of the pubis is rounded whereas a shallow depression is apparent on the plastron. The contact therefore appears to have been intermediate between the fully sutured condition seen in pleurodires and the loose articulation seen in cryptodires. The pubes are fused along the midline and form an expanded, ventrally curved, tongue-like epipubic process that is about as long as the remaining pubic body. The epipubic process is slightly discoloured relative to the main body of the pelvis, but it is unclear if it is calcified or ossified.

The exact nature of the ischial contact with the plastron is obscured by damage to the specimen, but it appears to have been more sutural than the pubic contact. The ischia contact the plastron along distally expanded processes that have a triangular cross-section, but it remains unclear if the ischia contact one another along the midline. A laminar piece of damaged bone is situated within the pelvic opening just posterior to the pubis. It is possible that this bone is a remnant of the ossified hypoischium, but too little is preserved to be confident in this identification (Figure 2B).

### Anterior plastral lobe

The anterior margin of the anterior plastral lobe is heavily damaged, but some insights are nevertheless available. The posterior entoplastral process is highly distinct in visceral view and extends far beyond the level of the axillary notches (not illustrated). The base of the dorsal epiplastral process sensu [31,32] is preserved on both sides of the specimen, but careful analysis of the ventral side of the carapace reveals that the dorsal epiplastral process did not articulate with the nuchal bone dorsally (see Additional file 1: Figure S1), as in *Proganochelys quenstedti*[31] and, perhaps, *Palaeochersis talampayensis*[33].

### Anterior vertebral column

The majority of bones that form the nuchal region of the shell are well preserved and show few signs of disarticulation (Figure 2C; Additional file 1: Figure S1C). The region consists of the posterior half of the seventh cervical vertebra, the entire eighth cervical vertebra, the first and second thoracic vertebrae, and the proximal portions of the eighth cervical rib and the first and second thoracic ribs.

The seventh cervical vertebra is only partially preserved and is strongly keeled ventrally. The eighth cervical vertebra is complete, but still partially embedded in matrix, and generally resembles those of other Triassic turtles [29,31,33]: it has a short centrum and a tall neural arch and dorsal process, is amphicoelous, the cervical ribs attach to a single transverse process that is located at the anterior third of the centrum, and a low keel decorates the ventral side of the centrum. There is no evidence of a formed articulation between the eighth cervical vertebra and the nuchal. The eighth cervical rib is damaged and its full length is therefore not apparent, but the portion that is preserved is about twice the anteroposterior length of the eighth cervical centrum. The eighth cervical rib has a single headed rib head and the body of the rib is round to oval in cross section for its entire preserved length. The eighth cervical rib was found in close alignment with the first thoracic rib posterior to the dorsal process of the scapulacoracoids (see Additional file 1: Figure S1).

The anterior central articulation of the first thoracic vertebra with the eighth cervical vertebra is oriented anteriorly, as in all basal turtles and pleurodires, and appears to be convex. The posterior articulation with the second thoracic vertebra is tight, but the suture is still apparent. The remaining thoracic vertebrae are still covered by sediment. Only the proximal portion of the first thoracic rib is preserved. It is a vertically oriented, recurved, flat element that articulates with the anterior end of the first thoracic vertebra proximally and has an elongate contact with the carapace dorsally. The vast majority of the distal portion of the first thoracic rib, however, appears to have been free, as seen in the few basal turtles that preserve this area (i.e., *Proganochelys quenstedti*[31] and *Heckerochelys romani*[34]). The second thoracic rib has an anteroposteriorly-broadened contact with the first and second thoracic vertebrae and is T-shaped in cross section. The anterior two-thirds of this contact is with the first thoracic vertebra, whereas the remaining third is with the second thoracic vertebra. The first and second thoracic vertebrae are lightly keeled.

### Sacral region

The sacral vertebrae and ribs are preserved in the posterior region of the specimen in addition to the posterior part of the last (tenth?) thoracic vertebra and large portions of the first to third caudal vertebrae (Figure 2B). The sacral vertebrae are tightly sutured to one another and with the last thoracic vertebra and lack a distinct ventral ridge. As in the majority of basal amniotes, the first sacral rib is significantly larger than the second [31]. The proximal end of the first sacral rib is anteroposteriorly expanded, much as the thoracic ribs are, but is unusual among turtles in that the anterior third of the rib contacts the last thoracic vertebra (only partially visible in Figure 2B). The first sacral rib is broadly expanded distally and suturally articulates with the ilium and with the second sacral rib. The proximal portion of the second sacral rib is also greatly expanded, but only has a small anterior contact with the first sacral vertebra (not visible in Figure 2). The left second sacral rib clearly articulates with the first sacral rib anteriorly and with the ilium distally, and appears to contact the carapace as well. The distal contact with the ilium is not apparent on the right side, but it is unclear if this is due to preservation. It is unclear if the thoracic ribs are involved in the formation of the sacrum, because the relevant area is covered by matrix, but the symplesiomorphic alignment of the ilium with the sacral ribs makes such a contact unlikely.

The two preserved caudal centra lack distinct ventral ridges. All caudals appear to be amphicoelous. The transverse processes of the first three caudal vertebrae are well developed and universally appear to be part of the vertebra, not separate ribs. The transverse processes have a broad base, are dorsoventrally flattened, and are slightly oriented to the anterior.

## Discussion

### The orientation of the scapula in basal turtles

The scapulacoracoid of extant turtles is a triradiate element consisting of the dorsal and acromion processes of the scapula and of the coracoid [31]. The dorsal process and the acromion process articulate dorsally and ventrally, respectively, along ligaments with the nuchal and the plastron and the scapulacoracoid can rotate along a vertical axis defined by these two flexible articulations. The entire shoulder girdle is therefore able to pivot along a vertical axis, allowing turtles to achieve greater stride length [35], a feature that is likely advantageous for any shelled organism.

The vertical orientation of the dorsal process in front of the ribcage was long believed to be a unique apomorphy of turtles, but a recent study demonstrates that this arrangement is universally found among basal amniotes [10]. Along those lines, a vertically oriented dorsal process is found in the potential stem turtle *Eunotosaurus africanus*[10], in the unambiguous stem turtle *Odontochelys semitestacea*[29], in the Late Triassic stem turtle *Palaeochersis talampayensis*[33], and can now be confirmed to be present in the oldest known turtle (i.e., amniotes with a fully developed turtle shell) *Proterochersis robusta*.

The scapulacoracoid of the Late Triassic *Proganochelys quenstedti* resembles that of *Proterochersis robusta* in all primary aspects, but has been described as having a dorsal process that is oriented obliquely towards the anterior [31]. The resulting, unusually shaped scapulacoracoid is difficult to fit inside the shell and cannot perform the rotating function seen in all other turtles as sometimes reconstructed [36] because it does not correctly articulate with the plastron. The vast majority of *P. quenstedti* specimens are plastically deformed and it is often difficult to assess the true shape of various bones. Among available specimens, the scapula is oriented anteriorly in some and vertically, as in *P. robusta*, in others [31]. However, the anterior orientation was favoured by Gaffney [31] in his final reconstruction of this taxon, because a single specimen, SMNS 16980, preserves this orientation on both sides of the skeleton and was therefore argued to be the least distorted. The observation that all newly described turtles that phylogenetically frame *P. quenstedti* have a vertically oriented scapula allows us to conclude that it is more likely that SMNS 16980 has symmetrically deformed scapulacoracoids and that the vertical orientation found in all other *P. quenstedti* specimens is the correct orientation for this taxon as well.

In addition to revealing that the scapular processes are oriented vertically in all basal turtles, the newly prepared specimen of *P. robusta* demonstrates that the acromion process was nearly in contact with the midline of the plastron (see Additional file 1: Figure S1). It is therefore apparent that the ability to rotate was well established in all Triassic turtles (i.e., amniotes with a fully developed turtle shell).

### The sutured pelvis of *Proterochersis robusta*

The morphology of the pelvis of the two groups of extant turtles differs fundamentally. In all extant cryptodires, the distal ends of all pelvic elements are narrow and lack any sutural connection with the shell. By contrast, in all extant pleurodires the distal ends of all pelvic elements are greatly expanded and more or less firmly sutured to the carapace dorsally and with the plastron ventrally.

We herein confirm that the pelvic elements of *Proterochersis robusta* are distally expanded and sutured to the shell, despite initial doubt from the senior author [14]. However, a significant difference is nevertheless present between the morphology of the sutured pelvis of all known unambiguous total group pleurodires and that of *P. robusta*: the sacrum of *P. robusta* is formed by the sacral ribs, whereas the sacrum of all known total group pleurodires is formed by the posterior thoracic ribs [12]. Despite this substantial structural difference we conclude that the primary homology [37] of the sutured pelvis of *P. robusta* and pleurodires is unproblematic, because a transition from one state to the other is feasible. In particular, given that the pelvis is normally associated with the two sacral vertebrae, it is highly plausible that the suturing of the pelvis occurred while the association with these vertebrae was maintained. Once the pelvis was sutured to the shell and the sacral vertebrae lost their primary function, it is plausible that the pelvis shifted anteriorly relative to the ribs and only then became associated with the thoracic vertebrae, while loosing its connection with the sacral vertebrae. However, even if the primary homology of the sutured pelvis is unproblematic and *P. robusta* is linked to pleurodires by the presence of a sutured pelvis, only a parsimony analysis using the total evidence available from the skeleton is able to test the secondary homology of this character [16,38,39].

### The phylogenetic placement of *Proterochersis robusta* and the age of the turtle crown

Although all recent phylogenies of turtle relationships are in agreement that homoplasy is rampant [12,14,25,26,28], some characters have proven to be less problematic and can be used to diagnose groups with confidence [19]. The sutured pelvis of *Proterochersis robusta* was already used in the type description to align this turtle with extant pleurodires [20], but numerous authors have since ignored the presence of the suture pelvis and preferred grouping *P. robusta* with other primitive turtles [40-42], likely because of the conspicuous presence of numerous primitive characters in this taxon, such as the presence of two pairs of mesoplastra, three pairs of inframarginal scutes, and an elongate posterior entoplastral process.

The cladistic revolution is the starting point for the modern debate. As all potential outgroups lack a sutured pelvis, Gaffney [21] concluded that the prolific presence of primitive characters in *P. robusta* is irrelevant and that this taxon should be grouped with extant pleurodires based on the shared derived presence of a sutured pelvis. However, this assessment was not tested rigorously for another 20 years until *P. robusta* was placed as a separate terminal taxon into a global matrix of turtle relationships. The first analysis do to so [22] arrived at the surprising conclusion that extant cryptodires and pleurodire share a number of derived characters that *P. robusta* lacks and that that taxon is therefore most parsimoniously interpreted as a stem turtle and the sutured pelvis a homoplasy. The majority of subsequent analyses agree on this interpretation [e.g., [14,22,24-26,34], but others still favour the pleurodiran affinities of this taxon [12,27,43].

Our morphological analysis of the new *P. robusta* specimen reveals a number of additional characters that further corroborate the basal placement of *P. robusta*, as they are present in basal turtles, but absent in both cryptodires and pleurodires. These include the 1) presence of a coracoid foramen, 2) bony ridges that connect the acromion process with the dorsal process, glenoid, and coracoid, 3) a short acromion process, 4) cervical ribs, and 5) elongate first thoracic ribs. Addition of these characters to those phylogenetic hypotheses that already advocate the basal placement of *P. robusta* is certain to further cement the placement of this taxon along the phylogenetic stem of crown Testudines.

**Figure 3:**
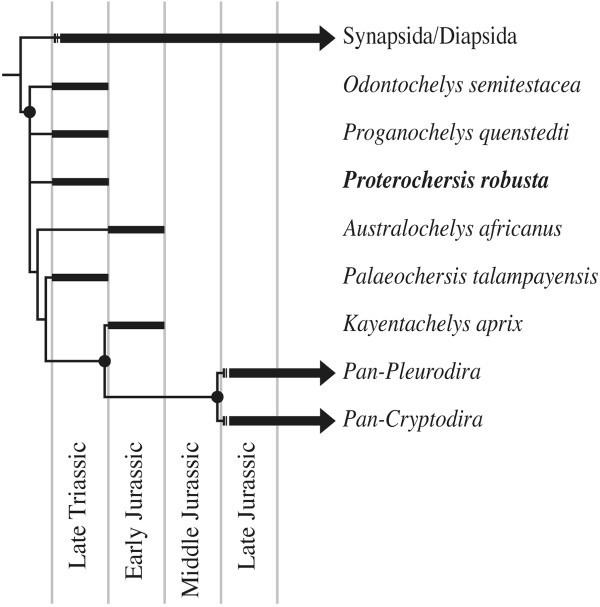
**Adams consensus tree of 30 most parsimonious trees resulting from the phylogenetic analysis presented herein.** Nodes highlighted with a circle are retrieved in the strict consensus topology as well.

To test the impact of the morphology of the girdles and vertebral column on those analyses that previously preferred the pleurodiran affinities of *P. robusta*, we herein chose to update the most recent and most carefully constructed character/taxon matrix [12] that favours this hypothesis. The matrix was primarily updated to reflect novel insights into the morphology of *P. robusta* and *K. aprix*[13,15,16] and by including the unambiguous proto-turtle *Odontochelys semitestacea*[29]. The parsimony analysis resulted in 30 most parsimonious trees (see Figure 3 for consensus cladogram) of 236 steps (consistency index excluding uninformative characters = 0.54; retention index = 0.74). The tree topology generally resembles that of Gaffney et al. [12], but differs in that *K. aprix* and *P. robusta* are universally placed outside of crown Testudines in all most parsimonious trees. The updated matrix therefore supports the basal position of these two taxa, implies that the sutured pelvis seen in *P. robusta* and pleurodires evolved independently, and is consistent with a basal divergence of crown turtles in the Middle Jurassic [14,19,24,26].

## Conclusions

Our study provides novel anatomical information for the oldest shelled turtle, which serves to help elucidate the numerous transformations necessary in the building of the unique turtle body plan. For example, the moderately robust shoulder girdle is intermediate in morphology between the more robust shoulder girdle found in basal amniotes and basal diapsids and the much more gracile, triradiate structure found in later turtles. The well-preserved specimen of *Proterochersis robusta* confirms that the shoulder girdle was situated vertical and anterior to the ribcage (as in *Odontochelys semitestacea*), indicating a similar condition was likely present in the slightly plastically deformed *Proganochelys quenstedti*, which is the plesiomorphic condition. Our study highlights the importance of cladistics in determining homology between structures. The pelvis of *P. robusta* and total group pleurodires is sutured to the shell (albeit with some important differences), but when analysed in a phylogenetic analysis it is revealed that this feature is actually homoplastic. This implies that the age of crown turtles is younger than some studies suggest and that *P. robusta* should not be utilized as a calibration point for molecular calibration studies [19]. Finally, our study shows the importance of fossils in evolutionary biology by providing insights into the acquisition of the novel testudinate body plan.

## Competing interests

The authors declare that they have no competing interests.

## Authors’ contributions

RRS initiated preparation of the specimen under study. WGJ documented specimen and performed phylogenetic analyses together with TRL. All authors helped interpret the data, draft the manuscript, and approved the final manuscript.

## Supplementary Material

Additional file 1: Figure S1 SMNS 17757, *Proterochersis robusta*, Late Triassic (Norian) Löwenstein Formation of Baden-Württemberg, Germany. **(A)** Oblique anteroventral view of shell lying on its dorsal side with plastron removed documenting the original position of both scapulacoracoids. Note that the coracoid blades are both arranged along a horizontal plane. **(B)** Left lateral view of left acromion and plastron. Note that the acromion process (below) almost contacts the plastron ventrally (above). **(C)** Ventral view of posterior nuchal area prior to the removal of the scapulacoracoids (compare with Figure 2). The ventral portions of the scapulacoracoids are removed to provide a better view of the area. Note that the dorsal process of the scapula is positioned in front of the eighth cervical rib and first thoracic rib. Also note that an attachment site is lacking for a dorsal epiplastral process. Click here for file

Additional file 2 Character Taxon Matrix. Click here for file
