# Supplementary figures and images for: The girdles of the oldest fossil turtle, Proterochersis robusta, and the age of the turtle crown
Source: BMC Evol Biol. 2013 Dec 6;13:266. doi: 10.1186/1471-2148-13-266 (PMC4077068; doi:10.1186/1471-2148-13-266)

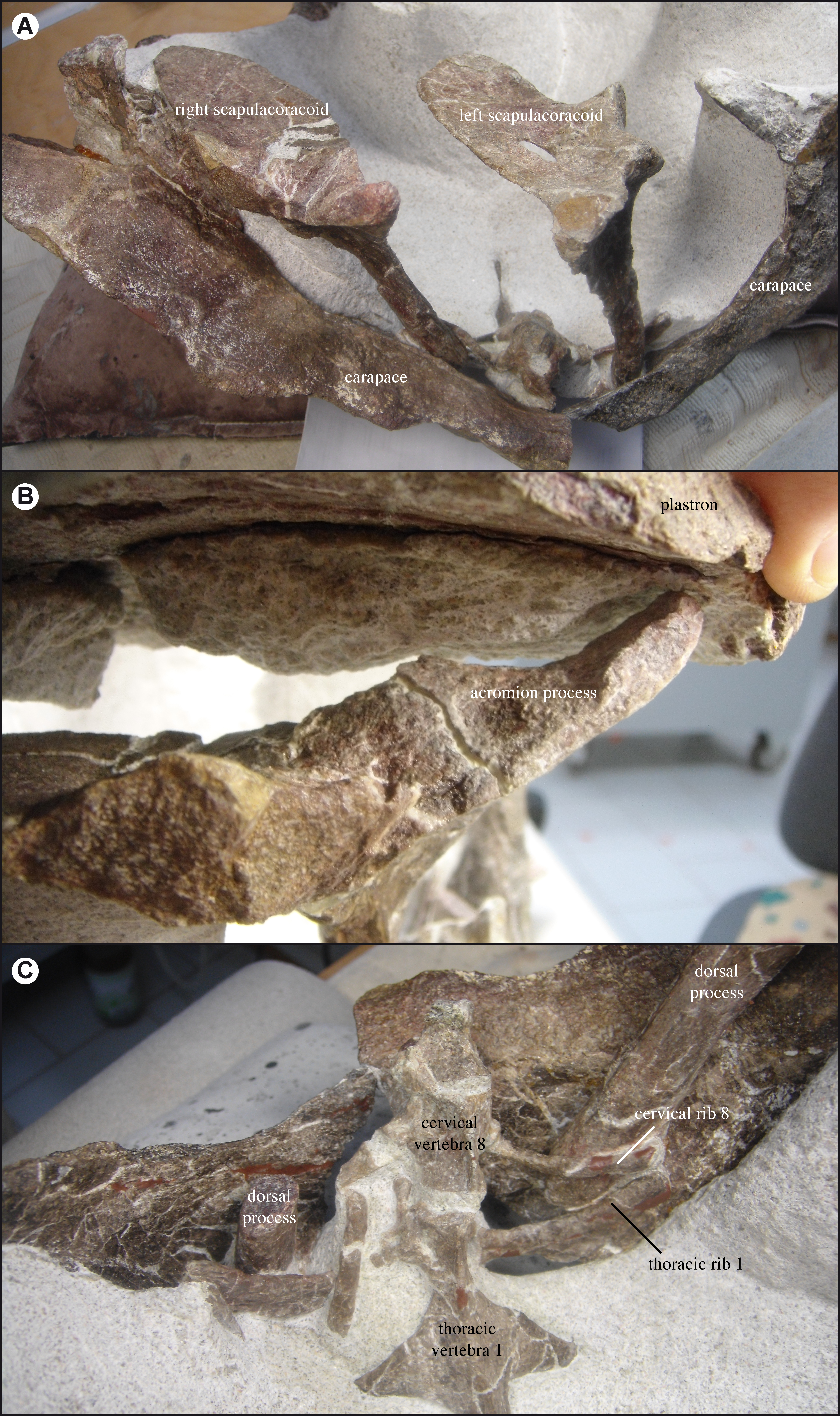

Supplement: Additional file 1: Figure S1 — SMNS 17757, Proterochersis robusta, Late Triassic (Norian) Löwenstein Formation of Baden-Württemberg, Germany. (A) Oblique anteroventral view of shell lying on its dorsal side with plastron removed documenting the original position of both scapulacoracoids. Note that the coracoid blades are both arranged along a horizontal plane. (B) Left lateral view of left acromion and plastron. Note that the acromion process (below) almost contacts the plastron ventrally (above). (C) Ventral view of posterior nuchal area prior to the removal of the scapulacoracoids (compare with Figure 2). The ventral portions of the scapulacoracoids are removed to provide a better view of the area. Note that the dorsal process of the scapula is positioned in front of the eighth cervical rib and first thoracic rib. Also note that an attachment site is lacking for a dorsal epiplastral process. [file 1471-2148-13-266-S1.tif]
